# Therapeutic Potential of Natural Antioxidants

**DOI:** 10.1155/2018/9471051

**Published:** 2018-08-15

**Authors:** Kota V. Ramana, Aramati B. M. Reddy, N. V. Ravi Kumar Majeti, Sharad S. Singhal

**Affiliations:** ^1^Department of Biochemistry and Molecular Biology, University of Texas Medical Branch, Galveston, TX 77555, USA; ^2^Department of Animal Biology, School of Life Sciences, University of Hyderabad, Hyderabad, TS 500046, India; ^3^Department of Pharmaceutical Sciences, Irma Lerma Rangel College of Pharmacy, Texas A&M University, College Station, TX 77843, USA; ^4^Department of Medical Oncology, Beckman Research Institute of City of Hope National Medical Center, Duarte, CA 91010, USA

Natural antioxidants are the plants- and other living organism-derived compounds with a strong potential to inhibit oxidative stress by controlling the formation of free radicals, scavenging the free radicals, interrupting the free radical-mediated chain reactions, and preventing the lipid peroxidation process. Thus, natural antioxidants have potential to balance the irregulated oxidative stress and to restore the cellular homeostasis. Therefore, natural antioxidants can decrease the deleterious effects of various oxidative stress-induced pathological conditions. The practice of using crude natural plant products to treat multiple diseases is ancestrally going on for thousands of years without or with the knowledge of their active antioxidant molecules well before the development of modern medicine with synthetic drugs and antioxidants. Indeed, after careful preclinical and clinical studies, most of the plant products have now been shown to act as potential therapeutic agents in preventing various human pathologies such as diabetes, aging, neurological, cardiovascular, and cancer. Furthermore, increase in the technical advancements made possible to identify vital active ingredients from the natural sources and develop them as potential therapeutic agents. Nowadays, an increase in the awareness of a healthy lifestyle has resulted in everyday consumption of nutritionally rich antioxidant organic natural food products over synthetic and processed foods which could restore our body's ability to fight against various kinds of stresses, infections, and related pathologies. Therefore, identification and development of novel antioxidants from nature could be beneficial in limiting the deleterious effects of oxidative stress in different pathological conditions. With this goal, we brought this special issue that reports the past and current research focused on identifying as well as understanding the mechanistic role of various antioxidants from the natural sources with a potential for future therapeutic development.

This special issue on “Therapeutic Potential of Natural Antioxidants” compiles 27 exceptional scientific manuscripts including eighteen research articles and nine review articles, which provide comprehensive evidence demonstrating the therapeutic significance of natural antioxidants in various pathological conditions.

The nine review articles of this issue describe the therapeutic potential of various natural antioxidants, possible mechanisms of action, and role in controlling oxidative stress-induced inflammatory signaling and pathologies. The review article by J. Kocot et al. nicely described the role of natural honey bees products specifically propolis, bee pollen, and royal jelly in counteracting the oxidative stress in various human diseases and mechanism of action. Further, in this excellent review article, authors have highlighted the influence of bee species, geographical location, and extraction methods on the composition of the bee product as well as their effectiveness as therapeutic agents. Similarly, S. Ahmed et al. in-depth discussed molecular mechanisms through which honey exhibits several medicinal and health benefits. Specifically, they discussed various cell signaling pathways that lead to apoptosis, proliferation, and regulation of inflammatory response. Another interesting review article by A. Baptista et al. has addressed antimicrobial and antioxidant properties of cashew (*Anacardium occidentale*), cajui (*Anacardium microcarpum*), and pequi (*Caryocar brasiliense*). This review described the analysis of data from a database of published studies on these compounds. Their report suggests that most of the studied species have shown significant antioxidant and antimicrobial properties.

The review article by M. Wojcik et al. described how antioxidative and anti-inflammatory properties of ancestrally well-known plant product curcumin beneficial for treating diabetes and cancer. In this article, authors have done an outstanding job in describing the current research on molecular signaling pathways through which curcumin exerted its anticancer and antidiabetic activities and discussed the necessity of its further therapeutic development.

Polyphenols naturally found in the fruits and vegetables have been shown to exhibit pleiotropic health benefits in preventing various human diseases such as diabetes, cancer, asthma, and cardiovascular. An excellent review article by S. Li et al. discussed how various polyphenols isolated from multiple natural sources help in the prevention of liver diseases. Further, they discussed the possible mechanisms of actions through which these natural polyphenols decrease liver diseases *in vitro* and *in vivo* animal models and clinical studies. Similarly, plant flavonoids with their potential antioxidative and anti-inflammatory actions prevent a number of diseases including diabetes, cancer, and cardiovascular. Recent investigations also suggest their beneficial role in depressive disorders. L. Hritcu et al. nicely reviewed various antidepressant flavonoids and their antioxidant potential. They further discussed the mechanism of action of flavonoids and how they combat the oxidative stress-induced depressive diseases. Ginseng is a natural plant product widely used in Asian countries for its anticancer and immunomodulatory activities. Q. Zheng et al. have discussed the role of ginsenoside Rb1, a major pharmacological extract from ginseng, in myocardial infarction. The review based on a meta-analysis of published information from scientific databases indicate that ginsenoside Rb1 possess cardioprotective actions in acute myocardial infarction largely due to its antioxidative, anti-inflammatory, and antiapoptotic properties and suggest that it could be an ideal candidate for further development for use in cardiovascular complications. In the same contest of cardiovascular complications, J. Tian et al. have discussed the role of various herbal medicines in diabetic cardiomyopathy. Specifically, they discussed the role and mechanism of actions of various natural flavonoids and others such as triptolide, curcumin, *Ginkgo biloba* extract, resveratrol, astragalus polysaccharides, *Salvia miltiorrhiza*, and ginseng. A final review article by X. Su et al. has described anticancer activities of sulforaphane, a natural antioxidant derived from cruciferous vegetables. Specifically, they discussed the role of sulforaphane in Nrf2 activation and its mediated antioxidative pathways. They have also described how this natural antioxidant reverses epigenetic alterations in cancers by targeting DNA methyltransferases, histone diacetyltransferases, and noncoding RNAs.

The research article by X. Liu et al. investigates the effect of isoliquiritigenin, a natural flavonoid compound derived from the herb licorice root, in the prevention of mild and acute pancreatitis in mouse models. Specifically, in caerulein-induced mild pancreatitis and L-arginine-induced acute pancreatitis mouse models, treatment of isoliquiritigenin shows significant beneficial effects. This study suggests that by inhibiting the oxidative stress and modulating the Nrf2/HO-1 pathways, isoliquiritigenin could prevent acute pancreatitis. Article by J. Benites et al. has reported anti-Helicobacter pylori activities of a series of 2- and 3-phenylaminojuglone-based substances prepared from juglone (extract of the husk of walnut fruit). They identified a most active compound 2-((3,4,5-trimethoxyphenyl)amino)-5-hydroxynaphthalene-1,4-dione 7 has potential antibacterial activity at low concentration. In another study by S.-H. Cha et al. reports protective effects of diphlorethohydroxycarmalol (DPHC), a polyphenol isolated from edible seaweed (*Ishige okamurae*) on methylglyoxal-induced oxidative stress in human embryonic kidney cells. They have also shown that DPHC increases the activation of Nrf2 transcription factor and expression of antioxidative enzymes. Further, DPHC also decreases the formation of advanced glycation end products and increases glyoxalase-1. These results suggest that DPHC could be developed further as potential therapeutic agent for the prevention of diabetic nephropathy.

A research report by I. Castellano et al. has demonstrated how anti-inflammatory activities of ovothiol A, a natural antioxidant isolated from sea urchin eggs, prevents hyperglycemia-induced endothelial dysfunction. They have shown that ovothiol A prevents TNF-alpha-induced monocyte adhesion to the human umbilical vascular endothelial cell (HUVEC), reactive oxygen, and nitrogen species and increases NO bioavailability. These results suggest a potential therapeutic use of this compound for the prevention of cardiovascular complications associated with the endothelial dysfunction. In the same lines, H. Sonowal et al. also reported how anti-inflammatory actions of Vialinin A, a p-terphenyl compound derived from Chinese edible mushroom *T. terrestris* and *T. vialis*, prevents endothelial dysfunction and neovascularization *in vitro* and *in vivo* models. Explicitly, they have shown that Vialinin A prevents VEGF-induced oxidative stress signals that activate NF-*κ*B mediated inflammatory response in HUVECs. They have also demonstrated that in a mouse model of Matrigel plug assay, Vialinin A prevents the formation of new blood vessels. These studies suggest that Vialinin A exhibits potent antioxidative and antiangiogenic properties.

Studies by A. Micali et al. demonstrated the protective effects of flavocoxid, a natural antioxidative flavonoid, on cadmium-induced renal toxicity. Specifically, they have shown that flavocoxid prevents cadmium-induced urea nitrogen and creatinine, iNOS, MMP-9, and pERK 1/2 expression. They have also demonstrated that this flavonoid preserved the glomerular and tubular structural and ultrastructural changes in cadmium-induced renal tissues in mice. These studies indicate protective effects of flavocoxid on cadmium-induced organ toxicities. The research article by Y. Xiang et al. investigates how quassinoid brusatol, isolated from the *Brucea javanica* plant, increases the chemotherapeutic efficacy of gemcitabine in pancreatic cancer. Brusatol prevents the growth of pancreatic cancer cells in culture as well as in nude mice xenograft models. Precisely, they have shown that brusatol inhibits Nrf2-mediated antioxidative pathways and increases reactive oxygen species (ROS). The results provide some evidence that brusatol could be used as an adjuvant therapy along with gemcitabine in preventing pancreatic cancer. Similarly, another study by L. Cheng et al. demonstrates that resveratrol increases the chemotherapeutic efficacy of gemcitabine in pancreatic cancer cells. This study indicates that resveratrol inhibits the expression of NAF-1 and increases ROS and Nrf2 signaling.

The article by H. Wu et al. determined the effect of resveratrol in promoting angiogenesis of endothelial progenitor cells (EPCs). They have shown that antioxidant actions of resveratrol suppressed the syndecan 4 shedding and increased tube formation of late EPCs via syndecan 4/Akt/eNOS pathway. M. Lee et al. reported the anti-inflammatory effects of black ginger (*Kaempferia parviflora*) on solar UV light irradiation-induced damage on mouse skin tissue. They have shown that this plant extract prevents UV-induced oxidative stress, expression of COX-2 and activation of MAPK signaling. This studies also found gallic acid, apigenin, and tangeretin as the major polyphenols in the extract. Another research study by R. Liu et al. demonstrated antioral cancer efficacy of melatonin. They have shown that melatonin inhibits the proliferation and apoptotic resistance of the oral cancer cells by regulating the ROS/Akt/cyclin D1/PCNA/Bcl-2/Bax signals. Another research article by D. Zhang et al. demonstrated a potent antioxidant activity of echinacoside, a phenylethanoid glycoside isolated from *Herba Cistanches*. In this study, BALB/c mice and HaCaT cells were pretreated with echinacoside followed by UVB exposure. They found that echinacoside prevents the skin injury, oxidative stress, DNA damage, and apoptosis caused by UVB exposure and also normalized the protein levels of ATR, p53, PIAS3, hnRNP K, PARP, and XPA. This report provides significance of this natural antioxidant in the prevention of UVB-induced DNA damage of the skin.

D. K. Yang and S.-J. Kim have reported protective effects of cucurbitacin I, a triterpenoid natural antioxidant, on hydrogen peroxide-induced H9c2 cardiomyoblast cytotoxicity. Specifically, this study demonstrates that cucurbitacin I prevents H_2_O_2_-induced oxidative stress, restores mitochondrial functions, and inhibits apoptotic response in H9c2 cells. Further, it also blocked the H_2_O_2_-induced activation of extracellular signal-regulated kinase 1/2, c-Jun N-terminal kinase, and p38 MAPK. This report provides an excellent evidence of how this natural antioxidant could exhibit a protective effect on oxidant-induced cardiac injury. L. Zhang et al. in their research article examined the effects of flavonoids extracted from *Citrus aurantium* in controlling the osteosarcoma progression. Results indicate that naringenin not naringin and hesperetin inhibited the growth of MG-63 cells which seems to be due to the potent antioxidant action of naringenin as compared to others. Most importantly, a small-scale clinical study using 95 osteosarcoma patients treated with naringenin showed a significant decrease in the osteosarcoma volume and their reoccurrence in a two-year follow-up. These studies provide concluding evidence for the therapeutic development of this natural antioxidant.

Another interesting research article by C. Guan et al. reports the immunosuppressive effect of antroquinonol, a ubiquinone derivative from the mushroom *Antrodia camphorata*, on CD8^+^ T cell proliferation and activation resist to depigmentation induced by hydrogen peroxide. They have antroquinonol that inhibits proliferation of CD8^+^ T cells by suppressing the production of cytokines IL-2 and IFN-*γ* and T cell activation markers CD69 and CD137 *in vitro*. Further, antroquinonol decreased hydrogen peroxide-induced CD8^+^ T cell infiltration in mice skin inhibited the production of IL-2 and IFN-γ and reduced the expression of CXCL10 and CXCR3. Thus, this study reports a novel immunomodulatory role of a natural antioxidant, antroquinonol. C. M. dos Santos et al. have investigated the chemical composition of the geopropolis of *Melipona quadrifasciata* anthidioides. They have shown antioxidant, antimutagenic, anti-inflammatory, and antimicrobial activities of these compositions. In another study, V. T. Chagas et al. also characterized the chemical composition of polyphenol-rich extracts of *Syzygium cumini* leaves. Specifically, they identified antidiabetic effects of these compounds in alloxan-induced diabetes in rats. Their results suggest that this plant extract could prevent antidiabetic activities by preventing the lipoxygenase activity and stimulating pancreatic insulin-secreting beta cells. Another study by U. P. dos Santos et al. characterized the lipophilic antioxidants and fatty acids from *Hancornia speciosa* leaves. Further, they examined the antidiabetic effects of ethanolic extract of *H. speciosa* extracts in diabetic Wister rats. Additionally, they have also examined microbiological quality and safety of *H. speciosa* leaves as well as antioxidant and antimutagenic activities.

It is evident from these papers that natural antioxidants play a major role in stabilizing the oxidative stress deregulated critical cellular pathways involved in the pathophysiology of a number of pathological disorders. Most of the articles reported a novel role of natural antioxidants in disease prevention and suggested potential future therapeutic development. The current change in the healthy lifestyle advocates search for more natural antioxidants with strong therapeutic potential which can eventually replace with some synthetic drugs with unwanted side effects. Thus, identification and development of natural antioxidants not only play an essential role in the prevention or therapy of human diseases but also interrupt any adversity that disrupts normal human health.

